# Selective modification of aligned carbon nanotubes by N_2_ plasma and their diode behavior

**DOI:** 10.1039/c8ra01396a

**Published:** 2018-03-16

**Authors:** Hsin-Jung Tsai, Yu-Ying Su, Chao-Chi Tseng, Wen-Kuang Hsu

**Affiliations:** Department of Materials Science and Engineering, National Tsing-Hua University Hsinchu 30013 Taiwan wkhsu@mx.nthu.edu.tw

## Abstract

Aligned carbon nanotubes made by pyrolysis of ferrocene are selectively treated with N_2_ plasma at various durations. Raman spectra reveal that polar bonds form at treated regions and result in increased Raman breathing mode. XPS support Raman and verify the C–O/C–N formations in carbon lattices. Electrical measurements show an unipolar p-type character as probes are placed in contact with untreated regions of nanotubes. Reposition of probes at untreated and treated regions respectively produces a current–voltage profile that resembles a p–n junction diode with forward current reaching a value as high as 100 mA at 3 V. *Ab initio* calculations confirm the on-tube junction and creation of a donor state is owing to C–N formations.

## Abstract 2

Fabrication of p–n junctions on aligned carbon nanotubes by N_2_ plasma.

## Graphical abstract

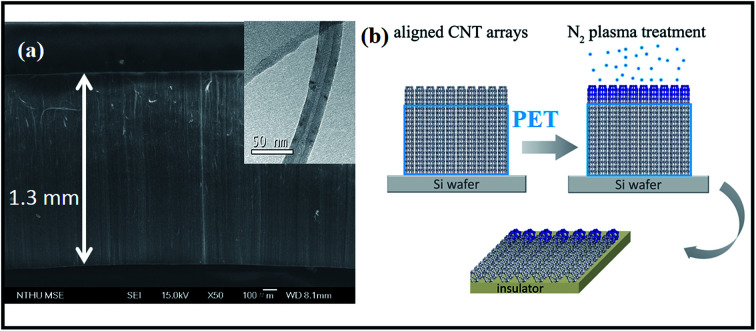

## 1. Introduction

Carbon nanotube (CNT) based field-effect transistors have drawn much attention in recent years and are believed to be capable of operating at a high speed and low energy loss.^1–3^ Diodes, on the other hand, also form the basis of logic circuits and consist of different components that allow current passage only one direction. At forward bias, current is limited by interfacial potential and then rises exponentially as voltage further increases. The reverse current however is severely restricted until breakdown voltage is reached. Accordingly, diodes are used for conversion of alternate into direct current as well as voltage stabilization. So far, only a few studies have demonstrated on-tube devices with current–voltage (*I*–*V*) profiles similar to diodes.^4,5^ For example, the introduction of pentagon–heptagon defects into carbon tubes results in unidirectional passage of electrical current.^5^ Hu *et al.* employ the controlled growth technique to generate a heterojunction which also yields an asymmetric *I*–*V* profile.^6^ However, nano-junctions are difficult to make on the regular basis, particularly electrical characterization is severely challenged by uncertainties arising from tube quality, thermal agitation, tube-matrix correlation and electrical contacts.^7,8^ In this work, a simple technique is developed to fabricate p–n junction along tube axis and processes involve selective-area modification of tube arrays by N_2_ plasma. Raman and XPS analyses reveal that C–N bonds form at regions near to array surfaces and behave as electron donors. Electrical tests show an unipolar p-type character as probes are placed in contact with untreated regions of tubes. Reposition of probes at untreated and treated regions establishes asymmetric *I*–*V* curves similar to diodes. Repeated tests confirm junction structure along tube axis and forward current is found to be as high as 100 mA.

## 2. Experimental

**Fig. 1:**
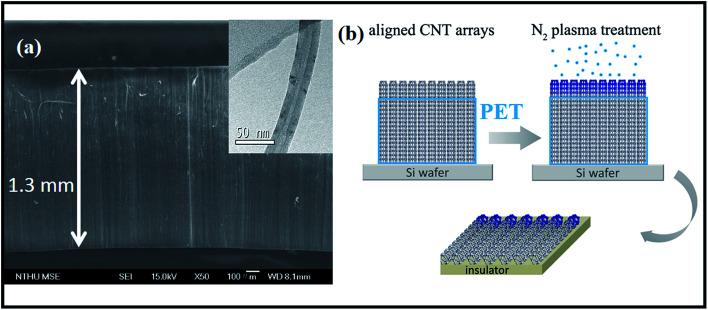
(a) SEM image of CNT array and (b) scheme of device fabrication. Insert: TEM image of individual CNTs.

Selective-area plasma treatments mean that chemical modification takes place at specific regions of nanotubes. In this respect, CNT sample must be made in an anisotropic fashion so operation can be accurately focused on area interested. A reported technique is here applied to produce micro-arrays that consist of aligned CNTs.^9^ First, a silicon wafer (30 × 30 mm) is placed in an electrical furnace and is repeatedly purged with N_2_ at 300 °C. Second, the furnace temperature is further promoted to 850 °C in the presence of N_2_ flow, followed by syringe injection of ferrocene–xylene solution (0.02 g/10 cm^3^) into wafer-containing furnace. Pyrolysis reaction produces dark film on wafer surfaces and SEM inspections confirm coatings to be vertically aligned CNTs in a compact form (Fig. 1a). Array has a dimension of 30 × 30 × 1.3 mm and the constituent tubes, according to TEM image, are multi-walled with diameter ranging at 20–35 nm (insert, Fig. 1a). Third, side-faces of tube array are covered with polyethylene terephthalate (PET) film. So only surface is exposed and is bombarded with 160 V ionized N_2_ plasma (400 sccm) for different durations (0.5, 1 and 4 min) (Fig. 1b). Fourth, treated arrays are carefully peeled off from substrate and are sliced into stripes (2 × 1 × 0.2 mm) for elemental analyses (Fig. 1b), including X-ray photoelectron emission spectroscopy (XPS, Perkin-Elmer, Model PHI1600) and selective-area of Raman (He–Ne laser, *λ* = 632.8 nm, scanning frequency = 100–2000 cm^−1^, HR800, HORIBA). *I*–*V* relation of treated array is probed in a high-resolution field emission SEM (Jeol, JSM-6500F) equipped with mobile-tungsten probe system and power supply (Keithley Model 4200-SCS). Since electrical conduction and chemical modification take place mostly at the outermost layers of tubes the *ab initio* calculation is therefore carried out using a single-walled tube as template. A zigzag tube (8,0) is built within a 5 × 5 nm window and one carbon atom is replaced by nitrogen to form amide and graphitic-N structures respectively. The density function theory (DFT) is treated by General Gradient Approximation (GGA) under exchange–correlation potential parametrization (PBE) of Perdew and Ultrasoft pseudopotentials.^10^ The SCF tolerance thresholds are set at 10^−6^ eV per atom and <0.04 Å^−1^ for structure convergence and the Monkhorst–Pack *k*-point grid separation. Since the built-in potential (*ϕ*_in_) is equivalent to band offset at p–n junction^11,12^ the band diagrams of tubes with and without doping are calculated on the DFT frame with a charged slab consisting of 80 atoms.^13,14^

## 3. Results and discussion

**Fig. 2:**
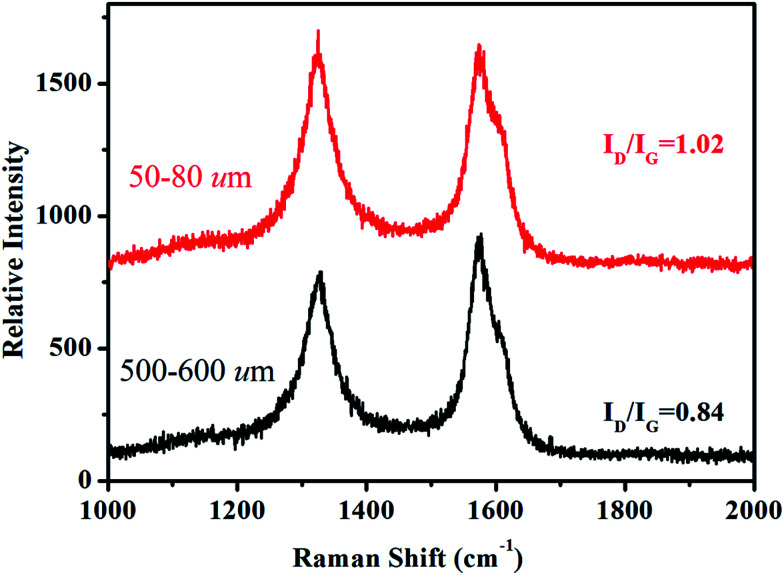
Selective-area Raman spectra of untreated (dark) and treated CNTs (red).

**Fig. 3:**
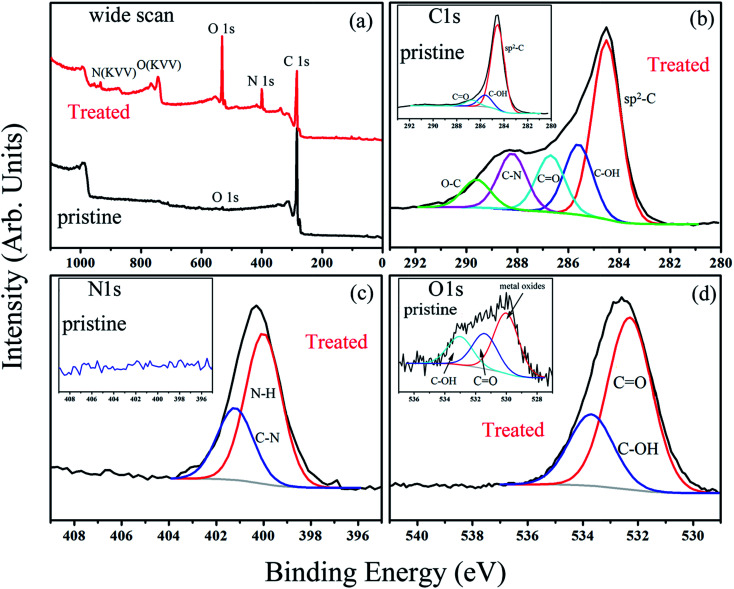
The wide-scan XPS spectra of pristine and treated CNTs (a), and deconvoluted spectra of C 1s (b), N 1s (c) and O 1s (d).

Depending on ionization power, the plasma induced chemical modification is usually limited to several Å for solid samples (*e.g.* Si).^15^ CNT arrays, however, are highly porous and therefore allow ionization to occur at a deeper range. Fig. 2 shows selective-area of Raman profiles obtained from regions that are 50–80 μm (red) and 500–600 μm below array surfaces (dark). Two peaks arising from C–C stretching (E_2g_, G-band, 1580 cm^−1^) and ring breathing (A_1g_, D-band, 1340 cm^−1^) are identified; the former is present in sp^2^ carbon and is independent of defect density. The latter, in contrast, is sensitive to bond length variation and has intensity as strong as G-band in defective tubes.^16^ Measurements give *I*_D_/*I*_G_ = 1.02 for upper and 0.84 lower parts and experiments carried out on similar regions confirm *I*_D_/*I*_G_(upper) > *I*_D_/*I*_G_(lower), indicative of chemical change mainly at array surfaces. XPS support Raman spectra, *i.e.* the breaking of hexagonal symmetry is due to chemical change. Fig. 3a shows XPS wide-scan spectra obtained from pristine and treated CNTs at 0–1100 eV. For untreated samples, spectra are dominated by conjugated C–C bonds (284.5 eV), along with a trace of oxygen possibly arising from carbonyl and hydroxyl (530.6 eV).^17^ Additional peak emerges as tubes are treated and lies at 400 eV, attributed to N 1s emission. Quantitative analyses based on peak integration give C = 96.4%, O = 3.6% before and C = 56.8%, O = 31.5% and N = 11.7% after treatments, supporting plasma induced functionalization. Fig. 3b shows spectral deconvolution of C 1s before (insert) and after treatments. First, the O 1s peak seen in Fig. 3a truly comes from carbonyl (C=O) and hydroxyl (C–OH). Second, treatments promote C=O and C–OH intensities based on the peak-to-peak ratio (O/C), accounting for increased O-content. Third, carboxylic (C–O) and C–N are also produced.

Various C–N groups have been verified in sp^2^ carbon, including pyridinic-N, pyrrolic-N, graphitic-N, amide, amine and lactam.^18^ Here deconvoluted N 1s spectra show two components in which the one at 401.2 eV fits well with graphitic-N (blue, Fig. 3c).^19^ The other associates with hydrogen and has a binding energy close to amide and lactam (399.9–400.2 eV).^20^ Both however form at defect edges and may coexist according to following. First, existing groups (hydroxyl and carbonyl) can react with nitrogen.^21^ In this case, network polarity increases and O 1s peaks move to high energy, consistent with a blue-shift of 0.8 eV in treated samples (insert & Fig. 3d).^18,19^ Second, carbonyl and hydroxyl are activated groups for electrophilic aromatic substitutions. Two factors however may account for the absence of pyridinic-N; (i) the ionization power here is too low to trigger pyridinic-N formation (>200 V);^22^ (ii) the pyridinic-N is changed into graphitic-N through atomic rearrangements.^23^

**Fig. 4:**
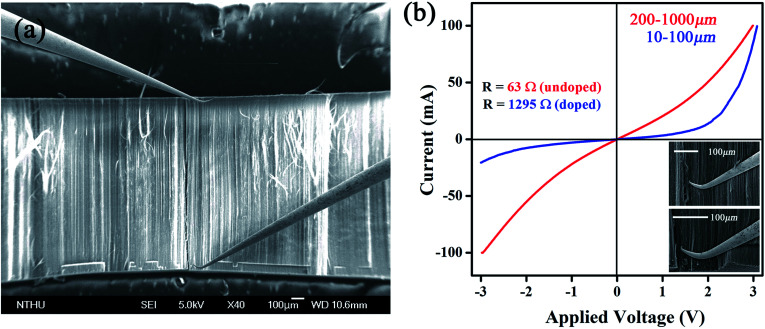
Two-probe system (a) and *I*–*V* curves obtained at various regions below surfaces (b). Electrical resistance obtained before and after treatments (top inserts) and zoom-in images taken at D and S locations (lower inserts).

Based on Raman and XPS, we believe that plasma induced modification takes place mostly at upper area and is further supported by *I*–*V* measurements. First, a tube array (1 × 3 × 3 mm) sliced from as-made bulk is placed in a SEM chamber (10^−6^ torr) for 1 h air evacuation. Second, two probes defined as S and D are crisscrossed to calibrate *I*–*V* relation at 0–100 mA/0–3 V. Third, the S is fixed at array base while D is brought in contact with area interested. It is worth mentioning in order to establish a good contact with tubes that probe tips are slightly bent to enlarge contact area and, according to SEM inspections, roughly 70–100 tubes lie on probing range (Fig. 4a). When D is placed at regions far below surfaces (*ca.* 400–1000 μm) the *I*–*V* profile is of unipolar p-type, consistent with previous reports on undoped CNTs (red, Fig. 4b).^8^ Reposition of D at regions near to array surface (10–100 μm) then creates a *I*–*V* curve that resembles a diode (blue). Repeated experiments verify that (i) the forward current (*I*_f_) is six orders of magnitude greater than value obtained from single tube devices, (ii) asymmetric transits into symmetric profile at *D* = 100–120 μm below array surface and, (iii) diode profile appears only at treated regions and is absent as D is placed at untreated area, excluding Schottky effect at metal/tube contacts. Additional evidence in support of diode effect comes from resistance (*R*) measurements before and after plasma treatments at ±1 V; the former yields 63 Ω and 1295 Ω for latter at ±1 V (top & lower inserts, Fig. 4).

**Fig. 5:**
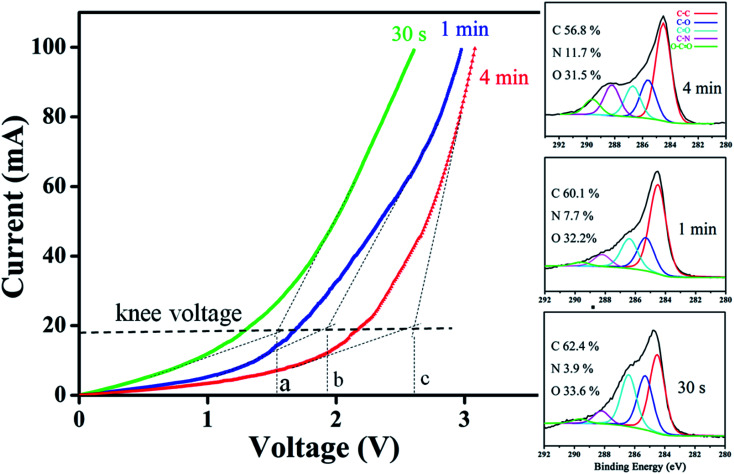
Variation of knee voltage with ionization times and corresponding XPS spectra.

**Fig. 6:**
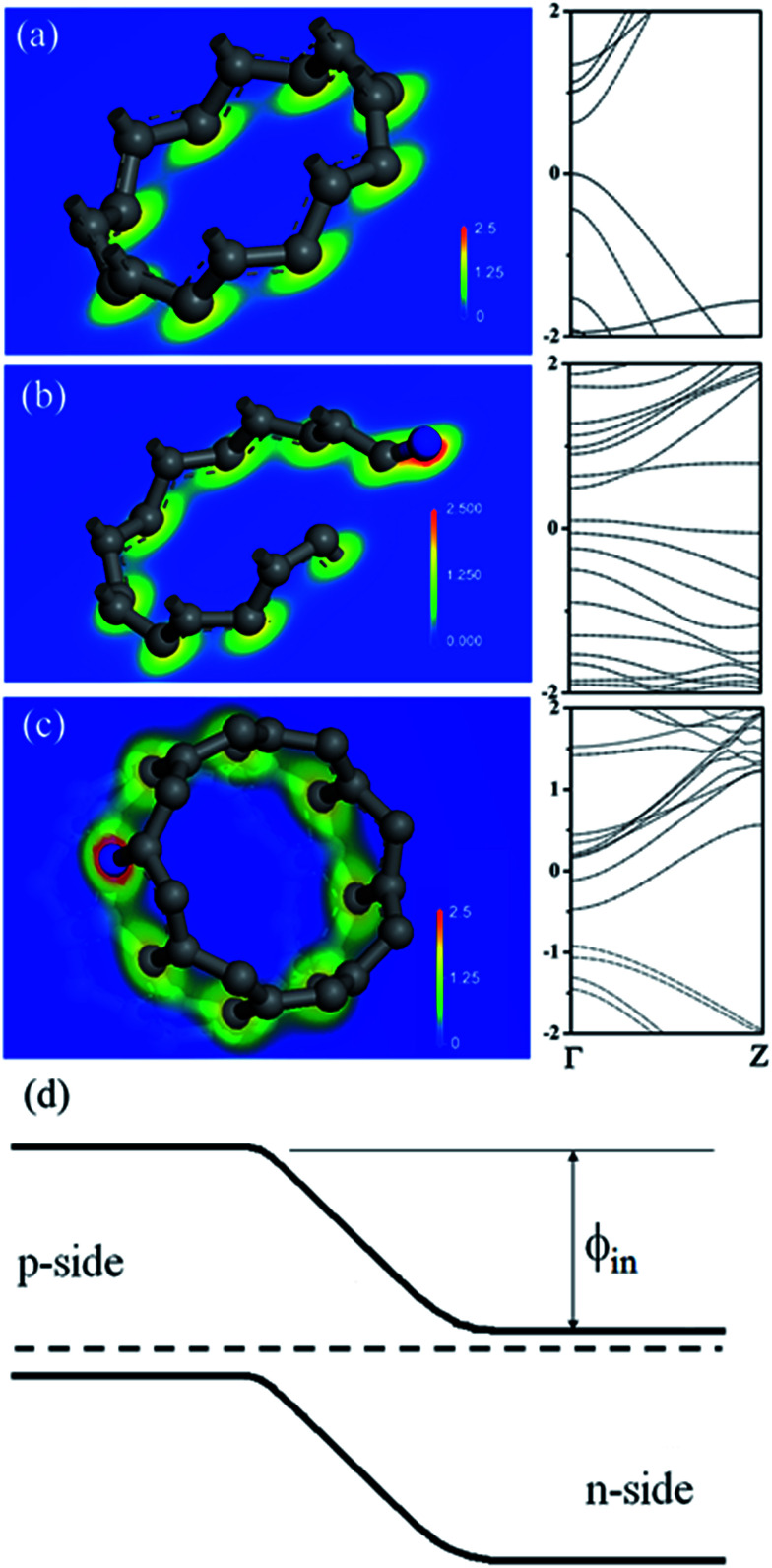
Mulliken charge distributions and band diagrams of (8,0) tube (a) and (8,0) tube doped with amide (b) and quaternary-N (c). Band offset and Fermi level equalization at the p–n junction (d). *ϕ*_in_ denotes built-in-potential and is calculated to be 0.603 eV for p–n_(amide)_ and 0.373 eV for p–n_(quaternary-N)_ junctions.

According to study, formations of either amide/amine or graphitic-N create donor states near to Fermi level (*E*_F_); the former donates electrons through σ-bonds known as inductive effect. Introduction of graphitic-N into carbon lattice, on the other hand, does not interrupt ring current and electron donation proceeds through π-resonance.^24^ For example, the energy of a 3 × 3 graphite doped with 5.5% N lies 1.21 eV above *E*_F_.^24^ Thermoelectric power verifies N-doped CNTs to be negative temperature dependence with conduction dominated by electrons.^25^ Here we find that diode characteristic truly associates with C–N formation and is verified by *I*–*V* plots against ionization durations (Fig. 5). For 0.5 min treatment, the N-content is 3.9% and knee voltage happens at 1.5 V (a), greater than undoped by 0.7 V (Fig. 4b). Diode character becomes pronounced as treatment is prolonged and measurements give N = 7.7% and knee voltage = 1.9 V (b) for 1 min and 11.7% and 2.6 V (c) for 4 min treatments. Electron donation from oxygenated groups is unlikely since O increase is small (1%) and C=O/CO_2_R behaves essentially as electron-withdraw. Fig. 6 shows band diagrams and simulations of undoped (a) in comparison with amide- (b) and graphitic-N containing tubes (c). For pure tube, charges distribute evenly on each atom and band gap (*E*_g_) between LUMO and HOMO is 0.623 eV. Substitutions, however, promote electron density around *E*_F_ and *E*_g_ due to emergence of π-state from N decreases to 0.42 eV and 0.35 eV for amide and graphitic-N. Note that charge density here is manifested by various colors (blue → red): *i.e.* the greater the density the deeper the reddish. In this respect, one can readily distinguish a greater density between atoms for graphitic-N substituted tube, supporting electron donation through π-bonds. Calculation further reveals the work function to be 4.6 eV for undoped and 4.2 eV and 4.5 eV for tube doped with 1.25% amide and graphitic-N respectively. Fig. 6d describes the band offset at interface in equilibrium; the *ϕ*_in_ being calculated to be 0.603 eV for p–n_(amide)_ and 0.373 eV for p–n_(graphitic-N)_. Again, *ϕ*_in(amide)_ > *ϕ*_in(graphitic-N)_ verifies origin of junction character mainly from amide substituents.

**Fig. 7:**
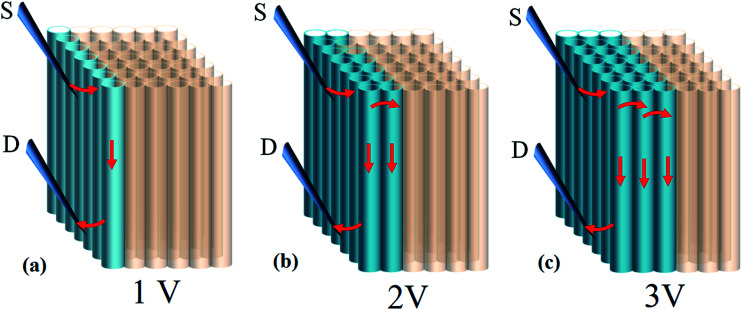
Intertube transfer mechanism at *V* > *V*_th_ which is proposed to account for high *I*_f_.

Question however remains as to why *I*_f_ can reach 100 mA while calculation based on parallel circuit model (*I*_*n*_ = *I*_1_ + *I*_2_ + *I*_3_…) yields only 0.7–1 μA where *n* denotes the number of tubes in contact with electrical probes (=70–100) and *I* is current carried by individual tubes (∼10 nA).^8^ Intertube transfer has been observed in tube aggregates and proceeds through hopping and tunneling; the former is of temperature dependence and displays a positive linear coefficients at *k*_B_*T* > T* where *k*_B_*T* and T* denote Boltzmann energy and crossover temperature.^7^ The latter remains to be justified and possibly occurs in electronically correlated tubes, *i.e.* a similar chirality.^26^ We believe that high *I*_f_ here also involves intertube mechanism. At low bias, transport takes place at tubes that are directly in contact with probes (Fig. 7a). As applied voltage is further promoted the kinetic energy of charges may surpass intertube barrier (*E*_a_). In this case, conduction extends to interior tubes and results in *n* increase (Fig. 7b and c). The threshold voltage (*V*_th_) for inter-transfer to occur can be estimated according to *V*_th_ = *E*_a(eV)_/*Q*_(*e*)_ where *E*_a_ lies 0.2–0.3 eV for compacted tubes at 300 K.^26^ The *Q* denotes elementary charge and equals to 4 *e* based on the two-band theory, *i.e.* each band transports 2 *e* (↑ & ↓).^27^ In practice, *Q* is strongly limited by lattice coupling/defects and ranges at 0.5–1 *e* based on report.^27^ Calculation then gives *V*_th_ = 0.2–0.6 V; value which is much lower than applied voltage (*i.e. V* > *V*_th_) and supports Fig. 7.

## 4. Conclusion

CNT arrays made by ferrocene pyrolysis are selectively treated with N_2_ plasma at different durations. In addition to oxygenated groups, the C–N bonds arising from amide/lactam and graphitic-N also form at treated regions and behave as electron donors. Electrical measurements display unipolar p-type (electron-deficit) profiles as both S and D are placed at untreated regions. Diode-like *I*–*V* curves emerge as D is repositioned at treated regions. Knee voltage increases with N-content and diode character mainly comes from amide substituents.

## Conflicts of interest

There are no conflicts to declare.

## Supplementary Material
